# The modified Si-Jun-Zi Decoction attenuates colon cancer liver metastasis by increasing macrophage cells

**DOI:** 10.1186/s12906-019-2498-4

**Published:** 2019-04-23

**Authors:** Jin-Yong Zhou, Min Chen, Cun-En Wu, Yu-Wen Zhuang, Yu-Gen Chen, Shen-Lin Liu

**Affiliations:** 1 The Affiliated Hospital of Nanjing University of Chinese Medicine, Jiangsu Province Hospital of Chinese Medicine, Nanjing, 210029 Jiangsu China; 2 Nanjing University of Chinese Medicine, Nanjing, 210023 Jiangsu China

**Keywords:** Modified Si-Jun-Zi decoction, Colorectal cancer, Metastasis, Macrophage cells

## Abstract

**Background:**

The modified Si-Jun-Zi Decoction (SJZ), a Chinese medicine formula, is clinically used against multiple malignancies including colorectal cancer (CRC). This study aims to evaluate the effect of modified SJZ on CRC liver metastasis and identify the therapeutic mechanisms.

**Methods:**

Human CRC cells with GFP fluorescence were transplanted into Balb/c nude mice spleens. Modified SJZ, 5-fluorouracil or the combined treatment was given for 3 weeks. CRC liver metastasis was measured by fluorescence imaging and plasma cytokines were analyzed. Furthermore, the effects of administration time and doses for the modified SJZ were investigated in nude mice.

**Results:**

Modified SJZ could increase the survival rate and reduce CRC liver metastasis in the nude mice model. Plasma GM-CSF level was elevated. Three weeks of treatment with the modified SJZ at the full dose (45 g/kg) could significantly increase the number of macrophages but not neutrophils in the spleen.

**Conclusions:**

These results indicate that modified SJZ can inhibit CRC liver metastasis by activating the innate immune system, providing a complementary and alternative therapy for CRC.

## Background

Colorectal cancer (CRC) is a common type of cancer, whose incidence ranks the third in Americans [1]. The highest incidence occurs in people aged 65~79 [2]. In China, CRC incidence has increased constantly during the past decades and the mortality rate of CRC is currently ranked the fifth among all cancers. It is estimated that around 191,000 patients died in the year of 2015 [3]. Metastasis of CRC is a biological feature of malignant tumor, accounting for most CRC patients’ death. The 5-year standardized survival rate ranges from 36 to 63% in Chinese CRC patients [4], which means that about half of these patients will experience CRC metastasis even after five years. Meanwhile it provides more time and opportunities for intervention. In CRC patients, the target organs of metastasis are mainly liver and lung [5]. However, there is still little knowledge about how to reduce metastasis.

In China, the modified Si-Jun-Zi Decoction (SJZ) is a famous traditional Chinese medicine (TCM) used against cancers including CRC in clinical treatment [6]. It is considered mild in nature and includes many tonic herbs such as *Codonopsis pilosula*, *Poria*, *Atractylodis Macrocephalae*, *Glycyrrhizae Radix et Rhizoma*. The SJZ Decoction has been claimed beneficial to the immune and gastrointestinal system in chronic disease patients [7, 8].

In clinical observations, SJZ treatment could increase 3-year and 5-year survival rates, and reduce metastasis-relapse rate in gastric cancer patients, compared with the chemotherapy group [9]. Modified SJZ treatment has been reported to restore humoral immune system and other researchers have observed an increase of T lymphocyte subsets after treatment in the patients with gastrointestinal tumors [10]. Furthermore, previous animal studies have shown that SJZ treatment could recover the hematopoietic system in irradiated mice [11] as well as enhance cellular and humoral immunity [12]. It has also been reported to counteract the cyclophosphamide-induced inhibition of peritoneal macrophage mediated cytotoxic activity [13]. Therefore, we hypothesized that this prescription may be beneficial to the innate immune system in the tumor-bearing body. However, its function on CRC liver metastasis and the underlying mechanism are still unclear.

In this study, we used a nude mice model of splenic–transplantation of colon cancer cells to study their liver metastasis. This model could partially mimic the pathogenesis of colon cancer, including cancer cells detaching from colon cancer tissue and entering the liver by portal venous system. This study aims to investigate the effect of modified SJZ on CRC liver metastasis in a mouse model and to explore its possible mechanism from the aspect of mice immune system.

## Methods

### Drug preparation

Modified SJZ was made in the Department of Pharmacy at Affiliated Hospital of Nanjing University of Chinese Medicine (Nanjing, Jiangsu, China). It is composed of *Radix Astragali* (15 g, Batch number: 1606066), *Codonopsis pilosula* (15 g, Batch number: 1607099), *Rhizoma Atractylodis Macrocephalae* (10 g, Batch number: 1606119), *Poria* (15 g, Batch number: 1605044), *Dioscoreae Rhizome* (15 g, Batch number: 1605034), *Glycyrrhizae Radix et Rhizoma* (5 g, Batch number: 1607086), *Mume Fructus* (10 g, Batch number: 1602071), *Sparganii Rhizoma* (10 g, Batch number: 1512060), *Curcumae Rhizoma* (10 g, Batch number: 1605130), *Agrimoniae Herba* (30 g, Batch number: 1604092), *patrinia* (30 g, Batch number: 1512157). They were all bought from Jiangyin Tianjiang Pharmaceutical Co., Ltd. (Jiangsu, China). All the granules of the above mentioned herbs were solved in 200 mL double-distilled water, heated at 120 °C, concentrated to 3 g herb/mL, and kept at 4 °C until usage.

### Cell line and reagents

The GFP-HCT-116 cell line (Retroviral GFP transduction) was purchased from Nanjing Origin Biotechnology Co. Ltd. (Nanjing, Jiangsu, China). Antibodies for flow cytometry were as follows: Mouse FcR Blocking (Miltenyi, 130–092–575), CD45-PE-Vio770 mouse (Miltenyi, 130–105-462), Anti-Mouse F4/80 Antigen PE (eBioscience, 12–4801-80), Anti-Mouse CD11b APC (eBioscience, 17–0112-81), Anti-Mouse Ly-6G-FITC (eBioscience, 11–9668-82).

### Animals and ethics statement

Forty eight Balb/c nu/nu mice, aged 4~6 weeks and weighted 20~24 g, were bought from Comparative Medicine Centre of Yangzhou University (Yangzhou, Jiangsu, China, animal certificate no. 0038475). The mice were kept in well ventilated sterile polypropylene cages in the SPF animal houses, free to sufficient sterilized water and complete formula feed, housed in a rodent facility at 25 °C with a 12 h light-dark cycle. All procedures involving animals and their care used were approved by the Animal Ethics Committee of Affiliated Hospital of Nanjing University of Chinese Medicine according to the Regulation of Experimental Animal Management (State Scientific and Technological Commission of the People’s Republic of China, number 2, 1988) and the Jiangsu Province Experimental Animal Management (Jiangsu province government, China, number 45, 2008). Experiments were started after acclimating for a week.

### Intrasplenic transplanted cancer cells

GFP-HCT-116 cells were cultured in 37 °C incubator with 5% CO_2_, harvested by digestion with 0.25% trypsin solution, washed twice with phosphate-buffered saline, and collected to a concentration of 1 × 10^8^/mL. We followed Giavazzi R’s method to intrasplenically transplant cancer cells [14]. Briefly, for anesthesia, animals were given an intraperitoneal injection of ketamine and xylazine. Each nude mouse was injected with 20 μL cell suspension in the spleen. They were recovered on a heating pad after surgery, till being returned to housing cages.

### Animal groups and treatment

The cancer cells transplanted nude mice were randomly divided into four groups: G1: negative control group, G2: 5-fluorouracil (5-Fu) group, G3: modified SJZ group and G4: the combined treatment group. Each group had 12 mice at the beginning. All treatments were given from the third day after transplantation and tumor formation was observed three weeks after injection [15]. The treatment of 5-Fu group was 15 mg/kg body weight, intraperitoneal injection, twice a week (the first day and the fourth day). The modified SJZ group treatment was 45 g/kg body weight, intragastric administration, twice a day.

Another forty Balb/c nude mice without tumor were prepared and randomly divided into control group, 1-week treatment group, 2-week treatment group, 3-week treatment group and 3-week half-dose treatment group. The modified SJZ treatment was give 45 g/kg mouse body weight, intragastric administration, twice a day, while half dose was once a day.

### Tumor imaging

Imaging was conducted according to previous literature [16]. Briefly, at the day of sacrifice, animals were euthanized according to NIH ARAC guidelines for euthanasia of rodents using carbon dioxide. Mice were euthanized by trained personnel via source of compressed gas in their home cage. After checking each mouse for lack of respiration and faded eye color, we confirmed their death. CO2 flow was maintained for a minute after respiration ceases. No anesthesia was used during the euthanasia.

All the mice were anatomically analyzed and images were acquired by a fluorescence stereo-microscope (model MZ650, Manjing Optic Instrument Inc., China) equipped with a D510 long-pass emission filter (Chroma Technology, Brattleboro, VT, USA) and a cooled color charge-coupled device camera (Qimaging, BC, Canada). Images were processed and analyzed by Image Pro plus 6.0 software (Media Cybernetics, Silver Spring, MD, USA).

### Animal plasma collection for cytokines arrays

Peripheral blood was collected from each mouse in the modified SJZ group and the control group at the time of sacrifice. Plasmas within the same group were pooled and tested by plasma cytokines analysis (Raybiotech, QAM-CYT-5) [17].

### Flow cytometry analysis of macrophages in mice treated with different time and dose of modified SJZ

At the end of experiment of mice without tumor, peripheral blood cells and spleen cells were collected and detected by flow cytometry. We used CD45, CD11b and F4/80 to identify macrophage cells and used CD45, CD11b and Ly-6G to identify neutrophils cells.

### Statistical analysis

Results were shown as mean ± standard error of mean, and statistical comparisons were made using one-way ANOVA, student’s *t* test or chi-square test. Significance was defined as *P*-value < 0.05.

## Results

### Modified SJZ increases the survival in the CRC mouse model

**Fig. 1:**
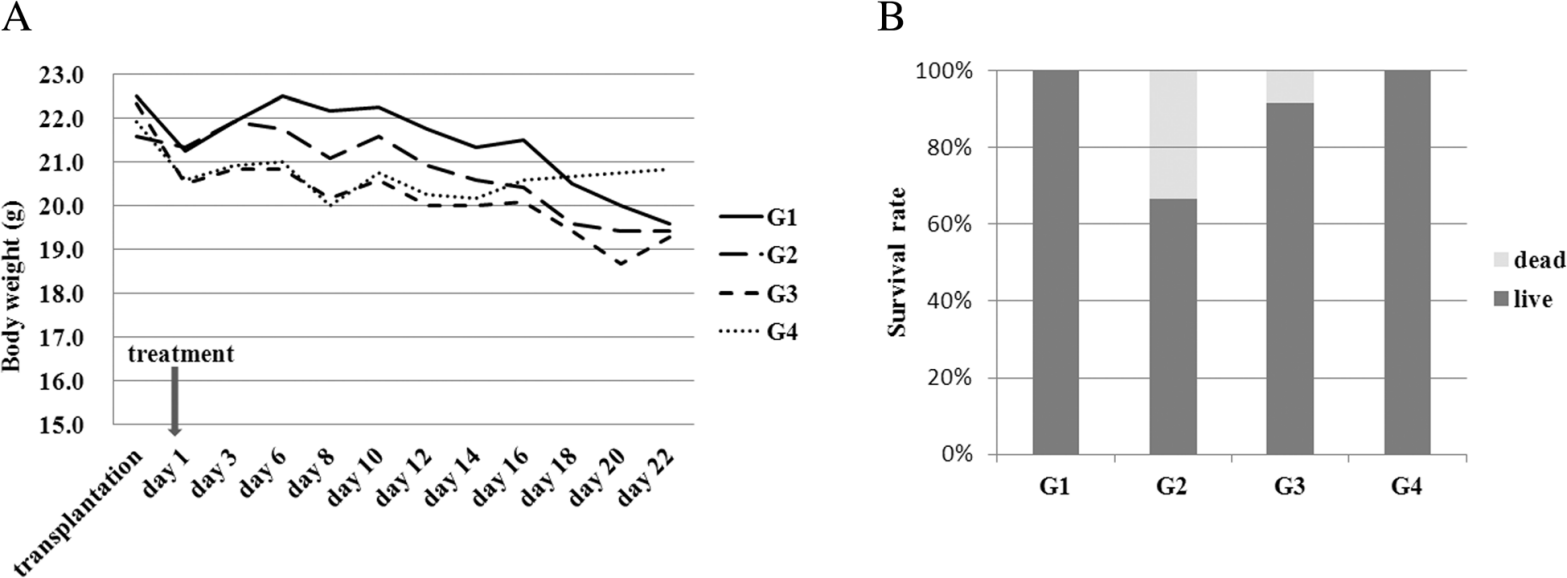
Changes of mice body weights and survival rates after 3 weeks of modified SJZ treatment. **a** Changes of mice body weights after treatments. G1:Control group (normal saline, intragastric administration twice a day for 3 weeks); G2:5-Fu group (15 mg/kg, intraperitoneal injection twice a week for 3 weeks); G3: MSJZ group (45 g/kg, intragastric administration twice a day for 3 weeks); G4: Combined (15 mg/kg 5-Fu, intraperitoneal injection twice a week and 45 g/kg MSJZ, intragastric administration twice a day for 3 weeks). Data are expressed as mean. **b** Changes of survival rates in different groups. 5-Fu: 5- fluorouracil; MSJZ: modified Si-Jun-Zi Decoction

We first examined the influence of modified SJZ on the survival rate of the nude mice. After three weeks of treatment, the average body weight decreased in all four groups (Fig. 1a). However, there was no statistically significant difference among the four groups at the time points before or after treatment. The survival rate was 66.7% (8/12) in 5-Fu group while 91.7% (11/12) in modified SJZ group (*p* = 0.047) (Fig. 1b). No mice died under the combined treatment. It indicates that the TCM may have effects on the vital organic system of the body, and therefore can increase survival.

### Modified SJZ decreased colon cancer liver metastasis in nude mouse model

**Fig. 2:**
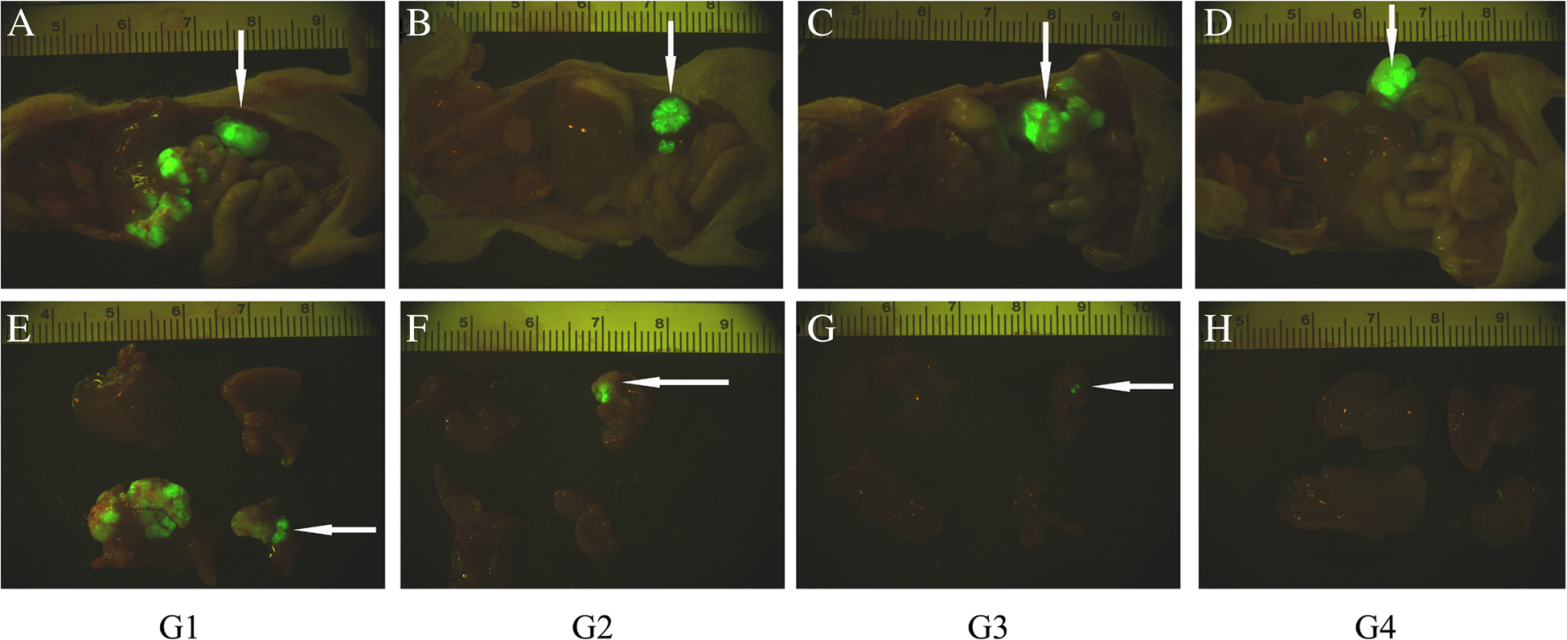
Representative images of colon cancer liver metastasis in different groups. **a**-**d**: Images of mice anatomy showing inner organs. The arrows pointing down indicated the transplanted tumor in the spleen. **e**-**h**: Images of mice livers and the arrows pointing left indicated the metastasis tumors. **a**, **e**: G1-Control group (normal saline, intragastric administration twice a day for 3 weeks); **b**, **f**: G2–5-Fu group (15 mg/kg intraperitoneal injection twice a week for 3 weeks); **c**, **g**: G3-MSJZ group (45 g/kg intragastric administration twice a day for 3 weeks); **d**, **h**: G4-Combined group (15 mg/kg 5-Fu, intraperitoneal injection twice a week and 45 g/kg MSJZ, intragastric administration twice a day for 3 weeks). 5-Fu: 5- fluorouracil; MSJZ: modified Si-Jun-Zi Decoction

After three weeks of treatment, the situation of colon cancer liver metastasis was examined by a fluorescence optical imaging system. As shown in Fig. 2, the green clusters indicate tumor tissues. Strict standard was adopted to decide if metastasis did not occur in a mouse, that is, there is no single green dot in the captured liver picture. When taking modified SJZ, metastasis rate dropped, from 90.9% (10/11, control group) to 44.4% (4/9, modified SJZ group), further to 30.0% (3/10, combined group), the latter two both having statistical differences against the control group (Table 1).

**Table 1:** Modified SJZ’s inhibitory effect on colon cancer liver metastasis

Group	Total^a^	Non-M^b^	M^b^	Metastasis rate	*P* value (v.s. control)
G1 (control)	11	1	10	90.9%	–
G2 (5-Fu)	7	2	5	71.4%	0.528
G3 (modified SJZ)	9	5	4	44.4%	0.024^*^
G4 (Combined)	10	7	3	30.0%	0.004^*^

^a^Those who have tumor in neither spleen nor liver were excluded

*P < 0.05 compared to control by chi-square test

^b^non-M:non-metastasis; M: metastasis

### Modified SJZ elevated GM-CSF expression in the peripheral blood of nude mice

Due to not reaching the necessary consuming volume of plasma by every single mouse, we pooled plasma within the same group for cytokines analysis. Among the cytokines, the level of GM-CSF increased in the treatment group (signal value 8489.14 vs. 4079.35). Besides, IFN-γ, IL-1α, and IL-3 increased (Table 2).

**Table 2:** Changes of cytokines in mice plasma after modified SJZ treatment (more than two folds)

Cytokines	Control	Modified SJZ	Modified SJZ/Control
GM-CSF	4079.35	8489.14	2.08
γ-IFN	6310.08	18,083.53	2.87
IL-1α	6312.31	12,652.31	2.00
IL-3	6329.8	15,174.82	2.40
IL-6	6046.64	14,167.83	2.34
IL-13	1787.22	3723.88	2.08
IL-17	6034.67	13,062.02	2.16
TARC	1277.37	2990.54	2.34

Data are expressed as fluorescence signal intensity. SJZ: Si-Jun-Zi Decoction. Modified SJZ/Control represents the ratio of fluorescence signal intensity of modified SJZ group to that of control group

### Modified SJZ increased the macrophage cells but not neutrophils in mice spleen

**Fig. 3:**
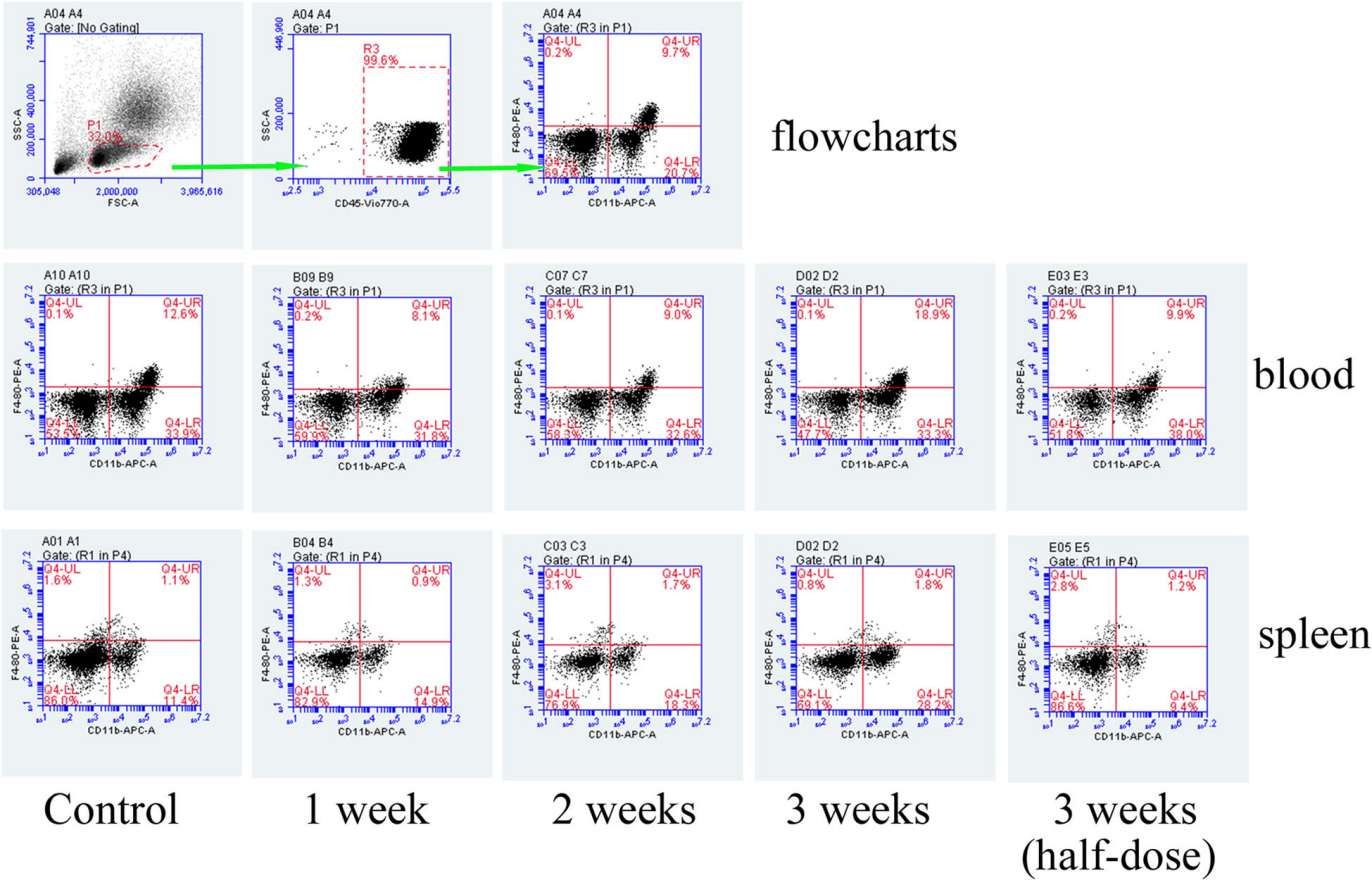
Representative flow cytometry results of macrophage cells in blood and spleen. The macrophage cells were shown as CD45 + CD11b + F4/80+ population (Q4-UR quadrant in each row). The percentage indicates CD45 + CD11b + F4/80+ cells in CD45+ cells in the monocyte and lymphocytes gate. Different times indicate the treatment times (45 g/kg intragastric administration twice a day for 1 week, 2 weeks and 3 weeks). Half-dose means 22.5 g/kg intragastric administration twice a day of modified Si-Jun-Zi Decoction

**Fig. 4:**
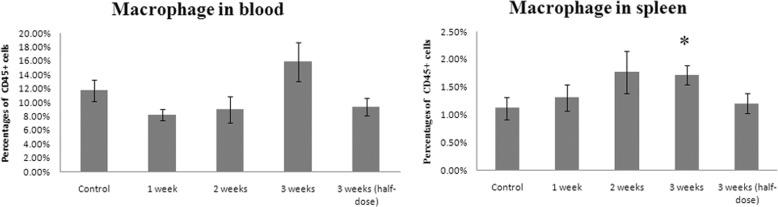
Statistic results of macrophage cells in blood and spleen. The time and dose treatment of modified Si-Jun-Zi Decoction was labeled under the columns. The data were shown as mean ± standard error of mean. *: *P* < 0.05 compared with control group by student *t* test

**Fig. 5:**
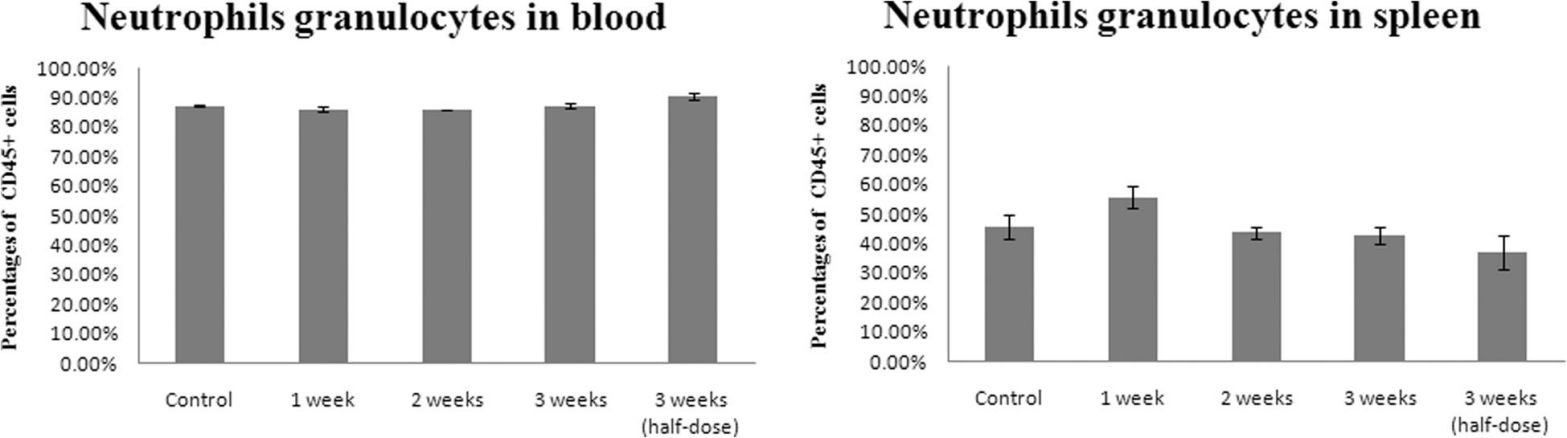
Statistic results of neutrophils granulocytes in blood and spleen. The time and dose treatment of modified Si-Jun-Zi Decoction was labeled under the columns. The data were shown as mean ± standard error of mean

At the end of experiment, we investigated changes of macrophage cells in mice peripheral blood and spleen by flow cytometry. Macrophage cells increased in number under 3-week treatment, compared to the control group, especially in the spleen (*P* < 0.05). However, it is noticeable that the 3-week half-dose treatment could not increase macrophage cell number effectively. On the other hand, treatment time less than three weeks has little effect on macrophage cells (Figs. 3 and 4). When it comes to neutrophils, no significant difference was observed among the groups (Fig. 5). It suggests that three weeks and full dose of modified SJZ treatment is necessary for macrophage cell proliferation.

## Discussion

In China, TCM plays a vital role in cancer prevention and treatment [18]. Recently, TCM has been commonly used as a supplementary therapy in the whole course instead of only the terminal stage of cancer [19]. TCM is known as a powerful tool to regulate human body’s immune system to maintain homeostasis based on ancient Chinese medicine theory [20, 21].

In this study, we found that modified SJZ was able to inhibit colon cancer liver metastasis. Modified SJZ could raise the survival rate after three weeks of treatment, alone or combined with 5-Fu treatment. Furthermore, TCM exerts its influence on innate immune system and enhanced certain plasma cytokines such as GM-CSF, IFN-γ, IL-1α and IL-3. When we carefully explored the time- and dose-response of the modified SJZ, we used the same time frame with the intrasplenic transplant of tumor cells in nude mice. Routinely, we examined 3 time points (1 week, 2 weeks and 3 weeks) and a half-dose group. Flow cytometry showed that the macrophage proportions were positively correlated to an increasing herb dose and treatment time.

At the beginning, we planned to use the orthotropic colon cancer mouse model, which would require us to transplant the colon cancer cells or tissues onto the colon surface to observe cancer metastasis by green fluorescence from cancer cells. In fact, the major shortcoming was that we could not easily exclude cancer cell contamination during the transplantation operation. However, liver metastasis mice model with spleen injected of colon cancer cells avoids the risk of cancer cell contamination because liver does not need to be touched. This model could partly mimic the clinic process of colon cancer. Meanwhile, fluorescence imaging provides us more sensitivity.

In our experiment, results showed that modified SJZ could increase GM-CSF in mice plasma. GM-CSF has been known as a powerful colony-stimulating factor which can stimulate proliferation of granulocyte and macrophage. Therefore, we focused on the innate immune system. Neutrophils are a kind of phagocyte in blood. At the initial stage of bacterial infection or some cases of cancer, they are one of the first responders migrating to inflammation sites [22, 23]. Macrophage cells also play a critical role in nonspecific defense because they can engulf and digest cellular debris, cancer cells, foreign substances and anything else that does not have certain types of surface proteins specific to healthy body cells. In this study, macrophage cell number elevation indicates that mice bodies have more capability to defend against cancer cells.

TCM has been reported to enhance the immune system. SJZ is a well-known formula that can modify immunity in humans. Tseng et al. found that GM-CSF secretion was significantly augmented when human peripheral blood monocytes were treated in vitro with 8 mg/ml SJZ for 18 h [8]. Huangqi injection, extracted from *Astragali Radix*, increased body weight and elevated white blood cell, monocyte, neutrophil, and lymphocyte levels of cyclophosphamide-treated mice [24]. Wang et al. also reported that the Shenqi Fuzheng Injection, composed of *Radix Codonopsis* and *Astragali Radix* and commonly used in TCM, improves immune function against chronic diseases. The injection treatment could accelerate spleen recovery index, peripheral white blood cell and bone marrow cell counts, enhance T cell and B cell proliferation responses, splenic nature killer cell activity and peritoneal macrophage phagocytosis in a dose-dependent manner [25]. Liu et al. found that the extract of *Codonopsis pilosula* maintained with sulfur fumigation could significantly increase white blood cell count and macrophage phagocytosis [26]. Another well-known TCM formula, Fufang e’jiao jiang, contains *Radix Codonopsis pilosulae* as one main ingredient. It was also reported to function in increasing the number of peripheral blood cells and bone marrow nucleated cells. It could increase GM-CSF and IL-3 level, and reduce TGF-β level in serum [27].

In this research, macrophages in the spleen of the modified SJZ group were elevated, indicating that the innate immune was activated by modified SJZ. Therefore, the promotion of macrophages may be a potential mechanism by which modified SJZ decreases the colon cancer liver metastasis rate. However, a significantly enhanced level of macrophage cells in the blood was not observed in the 3-week treatment compared to the control (15.85% ± 2.82% vs. 11.74% ± 1.53%, *P* = 0.21). One possible reason was that the macrophages may stay a steady status in blood while the spleen is a macrophage reservoir; another possible reason is that the increased macrophages were consumed by those detached cancer cells, which led to an overall no elevation. Nevertheless, more research is needed to investigate how modified SJZ activates the immune system, and to elucidate the functions of neutrophils and macrophages in the liver metastasis process of colon cancer. Administration time of modified SJZ also requires further research. Furthermore, TCM is thought to have multiple targets against CRC. Besides the immune system, it is worth exploring other targets involved in CRC liver metastasis.

## Conclusions

According the above results, modified SJZ was found to exert an inhibitory effect on colon cancer liver metastasis in vivo, alone or combined with 5-Fu. The potential mechanism may lie on its stimulation of cytokines such as GM-CSF to increase the number of macrophages in the spleen, and to clear colon cancer cells in the vascular system in time. This finding indicates that the TCM may target the innate immune system among its multiple targets.

## Abbreviations

5-Fu: 5- fluorouracil
CRC: Colorectal cancer
GFP: Green fluorescent protein
GM-CSF: Granulocyte-macrophage colony-stimulating factor
SJZ: Si-Jun-Zi Decoction
TCM: traditional Chinese medicine

## References

[1] Siegel RL, Miller KD (2017). Cancer statistics, 2017. CA Cancer J Clin.

[2] Siegel RL, Miller KD, Fedewa SA, Ahnen DJ, Meester RGS, Barzi A, Jemal A (2017). Colorectal cancer statistics, 2017. CA Cancer J Clin.

[3] Chen W, Zheng R, Baade PD, Zhang S, Zeng H, Bray F, Jemal A, Yu XQ, He J (2016). Cancer statistics in China, 2015. CA Cancer J Clin.

[4] Sankaranarayanan R, Swaminathan R, Brenner H, Chen K, Chia KS, Chen JG, Law SC, Ahn YO, Xiang YB, Yeole BB, Shin HR, Shanta V, Woo ZH, Martin N, Sumitsawan Y, Sriplung H, Barboza AO, Eser S, Nene BM, Suwanrungruang K, Jayalekshmi P, Dikshit R, Wabinga H, Esteban DB, Laudico A, Bhurgri Y, Bah E, Al-Hamdan N (2010). Cancer survival in Africa, Asia, and Central America: a population-based study. Lancet Oncol.

[5] Slesser AA, Georgiou P, Brown G, Mudan S, Goldin R, Tekkis P (2013). The tumour biology of synchronous and metachronous colorectal liver metastases: a systematic review. Clin Exp Metastasis.

[6] Xiao H, Yang J (2011). Immune enhancing effect of modified sijunzi decoction on patients with colorectal cancer undergoing chemotherapy. Zhongguo Zhong Xi Yi Jie He Za Zhi.

[7] Cai J, Wang H, Zhou S, Wu B, Song HR, Xuan ZR (2008). Effect of Sijunzi decoction and enteral nutrition on T-cell subsets and nutritional status in patients with gastric cancer after operation: a randomized controlled trial. Zhong Xi Yi Jie He Xue Bao.

[8] Tseng J, Li TL (1996). Si-Jun-zi-tang regulate granulocyte macrophage colony-stimulating factor secretion by human peripheral blood mononuclear cells. Am J Chin Med.

[9] Wu B, Xuan ZR (2007). Progress in research on applying Sijunzi decoction in treating digestive malignant tumor. Chin J Integr Med.

[10] Liang C, Zhang SH, Cai ZD (2005). Effects of early intestinal application of sijunzi decoction on immune function in post-operational patients of gastrointestinal tumor. Zhongguo Zhong Xi Yi Jie He Za Zhi.

[11] Hsu HY, Yang JJ, Lian SL, Ho YH, Lin CC (1996). Recovery of the hematopoietic system by Si-Jun-Zi-tang in whole body irradiated mice. J Ethnopharmacol.

[12] Xu AH, Gong YX, Gu WR, Wang XW (1993). Comparison of the effect of sijunzi decoction, siwu decoction and bazhen decoction on immune function in mice. Zhongguo Zhong Yao Za Zhi.

[13] Dou J, Wu MY (1990). Effect of si Jun zi tang on the macrophage cytotoxic activity in mice. Zhong Xi Yi Jie He Za Zhi.

[14] Giavazzi R, Garofalo A, Damia G, Garattini S, D’Incalci M (1988). Response to flavone acetic acid (NSC 347512) of primary and metastatic human colorectal carcinoma xenografts. Br J Cancer.

[15] Lee M, Price D, Specht S, Stemmler N, Katoh A (1992). Interferon modulation of 5-fluorouracil: use in neoadjuvant therapy inhibits experimental liver metastases in nude mice. Anti-Cancer Drugs.

[16] Jin H, Yang Z, Wang J, Zhang S, Sun Y, Ding Y (2011). A superficial colon tumor model involving subcutaneous colon translocation and orthotopic transplantation of green fluorescent protein-expressing human colon tumor. Tumour Biol.

[17] Correnti JM, Cook D, Aksamitiene E, Swarup A, Ogunnaike B, Vadigepalli R, Hoek JB (2015). Adiponectin fine-tuning of liver regeneration dynamics revealed through cellular network modeling. J Physiol.

[18] Lin H, Liu J, Zhang Y (2011). Developments in cancer prevention and treatment using traditional Chinese medicine. Front Med.

[19] Qi F, Zhao L, Zhou A, Zhang B, Li A, Wang Z, Han J (2015). The advantages of using traditional Chinese medicine as an adjunctive therapy in the whole course of cancer treatment instead of only terminal stage of cancer. Biosci Trends.

[20] Wang JH (2012). Traditional Chinese medicine and the positive correlation with homeostatic evolution of human being: based on medical perspective. Chin J Integr Med.

[21] Meng MB, Wen QL, Cui YL, She B, Zhang RM (2011). Meta-analysis: traditional Chinese medicine for improving immune response in patients with unresectable hepatocellular carcinoma after transcatheter arterial chemoembolization. Explore (NY).

[22] Waugh DJ, Wilson C (2008). The interleukin-8 pathway in cancer. Clin Cancer Res.

[23] De Larco JE, Wuertz BR, Furcht LT (2004). The potential role of neutrophils in promoting the metastatic phenotype of tumors releasing Interleukin-8. Clin Cancer Res.

[24] Qu T, Li Z, Zhao S, Li A, Qin X (2016). A metabonomic analysis reveals novel regulatory mechanism of Huangqi injection on leucopenia mice. Immunopharmacol Immunotoxicol.

[25] Wang J, Tong X, Li P, Cao H, Su W (2012). Immuno-enhancement effects of Shenqi Fuzheng injection on cyclophosphamide-induced immunosuppression in Balb/c mice. J Ethnopharmacol.

[26] Liu CS, Wang YP, Shi YB, Ma XM, Li HL, Zhang XY, Li ST (2014). Effect of Codonopsis Radix maintained with sulfur fumigation on immune function in mice. Zhong Yao Cai.

[27] Liu M, Tan H, Zhang X, Liu Z, Cheng Y, Wang D, Wang F (2014). Hematopoietic effects and mechanisms of Fufang e'jiao jiang on radiotherapy and chemotherapy-induced myelosuppressed mice. J Ethnopharmacol.

